# Effectiveness of a smartphone application for improving healthy lifestyles, a randomized clinical trial (EVIDENT II): study protocol

**DOI:** 10.1186/1471-2458-14-254

**Published:** 2014-03-15

**Authors:** José I Recio-Rodríguez, Carlos Martín-Cantera, Natividad González-Viejo, Amparo Gómez-Arranz, Maria S Arietaleanizbeascoa, Yolanda Schmolling-Guinovart, Jose A Maderuelo-Fernandez, Diana Pérez-Arechaederra, Emiliano Rodriguez-Sanchez, Manuel A Gómez-Marcos, Luis García-Ortiz

**Affiliations:** 1 The Alamedilla Health Center, Castilla y León Health Service, USAL, IBSAL, Salamanca, Spain; 2 Primary Health care Research Unit of Barcelona, Primary Healthcare University Research Institute IDIAP-Jordi Gol, Barcelona, Spain; 3 Torre Ramona Health Center, Aragón Health Service, Zaragoza, Spain; 4 Casa de Barco Health Center, Castilla y León Health Service, Valladolid, Spain; 5 Primary Care Research Unit of Bizkaia, Basque Health Service-Osakidetza, Bilbao, Spain; 6 Río Tajo Health Center, Castilla-La Mancha Health Service, University of Castilla-La Mancha, Talavera de la Reina, Spain; 7 EVIDENT Group. redIAPP: Red de Investigación en Actividades Preventivas y Promoción de la Salud (Research Network on Preventive Activities and Health Promotion), Salamanca, Spain

**Keywords:** Physical activity, Food, Information and communication technologies, Arterial aging

## Abstract

**Background:**

New technologies could facilitate changes in lifestyle and improve public health. However, no large randomized, controlled studies providing scientific evidence of the benefits of their use have been made. The aims of this study are to develop and validate a smartphone application, and to evaluate the effect of adding this tool to a standardized intervention designed to improve adherence to the Mediterranean diet and to physical activity. An evaluation is also made of the effect of modifying habits upon vascular structure and function, and therefore on arterial aging.

**Methods/Design:**

A randomized, double-blind, multicenter, parallel group clinical trial will be carried out. A total of 1215 subjects under 70 years of age from the EVIDENT trial will be included. Counseling common to both groups (control and intervention) will be provided on adaptation to the Mediterranean diet and on physical activity. The intervention group moreover will receive training on the use of a smartphone application designed to promote a healthy diet and increased physical activity, and will use the application for three months. The main study endpoints will be the changes in physical activity, assessed by accelerometer and the 7-day Physical Activity Recall (PAR) interview, and adaptation to the Mediterranean diet, as evaluated by an adherence questionnaire and a food frequency questionnaire (FFQ). Evaluation also will be made of vascular structure and function based on central arterial pressure, the radial augmentation index, pulse velocity, the cardio-ankle vascular index, and carotid intima-media thickness.

**Discussion:**

Confirmation that the new technologies are useful for promoting healthier lifestyles and that their effects are beneficial in terms of arterial aging will have important clinical implications, and may contribute to generalize their application in favor of improved population health.

**Trial registration:**

Clinical Trials.gov Identifier: NCT02016014

## Background

### Benefits of physical activity

There is abundant scientific evidence of the biological and psychological benefits of physical activity [1]. On the other hand, low cardiopulmonary fitness is associated with an increased risk of cardiovascular morbidity-mortality, while improved physical fitness is associated with a decrease in mortality risk [2]. The results of a metaanalysis on the influence of physical activity upon blood pressure indicate a mean reduction of 3.8 mmHg in systolic blood pressure (SBP) and 2.6 mmHg in diastolic blood pressure (DBP) [3]. Aerobic exercise could attenuate arterial hardening as observed in the Baltimore Longitudinal Study, in which male athletes had a lesser pulse wave velocity (PWV), augmentation index (AIx) and SBP than sedentary individuals [4]. Improvement in carotid artery elasticity has also been recorded in previously sedentary subjects who started a physical exercise program [5]. Aerobic exercise has been related to a decrease in atherosclerosis progression in humans [6]. The results of the first phase of the EVIDENT study [7] have demonstrated an association between physical exercise and improved vascular structure and function [8]. An association has also been reported between the time people spend seated and mortality. However, the results are even more relevant on combining the time spent seated (6 hours a day) with low physical activity (< 24.5 METS/hours/week), yielding a relative risk (RR) of 1.94 (95% confidence interval (95% CI): 1.70-2.20) for women and 1.48 (95% CI: 1.33-1.65) for males, as opposed to those who spend less time seated and have greater activity [9]. The EVIDENT study has also found increased arterial stiffness to be related to the number of hours spent watching television [10]. On the other hand, physical activity shows an inverse correlation to the concentrations of fibrinogen [11].

### Interventions for increasing physical activity

Although there is abundant evidence of the benefits of physical activity, the percentage of individuals who may be considered active is low in our setting [12]. In the United Kingdom, the prevalence of sedentarism reaches 60% in males and 74% in females [13], while in Spain the PEPAF (Experimental Program for Physical Activity Promotion) recorded a 75% sedentarism rate [14]. Different methods have been used in an attempt to promote physical activity, though with conflicting results. A Cochrane review concluded that there are no data to support the hypothesis that multiple-component community-based procedures are effective in increasing the physical activity of the population [15]. A recent metaanalysis of studies in the primary care setting has concluded that the promotion of physical activity in sedentary subjects increases the number of individuals who are classified as active subjects after 12 months (odds ratio (OR): 1.42; 95% CI: 1.17-1.73), though the effect has not been demonstrated over the long term [16,17]. The PEPAF study intervention resulted in an increase in physical activity versus the controls of 18 min./week (95% CI: 6–31 min./week), with a rise in METS/hours/week of 1.3 [95% CI: 0.4-2.2]. The proportion of individuals reaching the recommended minimum physical activity was 3.9% greater in the intervention group (1.2% to 6.9%) than in the control series. Few randomized, controlled clinical trials have evaluated the impact of interventions in terms of a decrease in sitting time, though an intervention designed to promote walking while working was able to reduce the daily sitting time by 21 minutes [18]. Evidence is even more limited in populations with chronic diseases, though an intervention in type 2 diabetics has recorded a reduction in daily sitting time of 23 minutes [19].

### Benefits of the Mediterranean diet

The Mediterranean diet is presently regarded as the model of most healthy diet. It reflects the traditional eating habits of countries such as Greece, southern Italy and Spain. The main characteristics of this diet are: a) important consumption of cereals, legumes, nuts, fruit and vegetables; b) use of olive oil for cooking purposes and for the dressing of salads and vegetables (representing the main source of fat in the form of monounsaturated fatty acids); c) moderate to high consumption of fish; d) moderate to low consumption of chicken and dairy products; e) low consumption of red meats and meat products; and f) moderate alcohol consumption, mainly in the form of red wine with meals [20]. There is convincing evidence that the Mediterranean diet may have a positive effect upon endothelial function [21] and can lower peripheral blood pressure [22]. A correlation has also been established with vascular structure, as assessed by carotid intima-media thickness [23], as well as with decreased mortality due to any cause [24] and, more specifically, due to coronary disease [25]. The results of the EVIDENT I study found obese males to show the poorest adherence [26].

### Interventions for improving eating habits (Mediterranean diet)

Few intervention studies with the Mediterranean diet have been carried out. A randomized clinical trial (the Lyon Diet Heart Study) [27], involving a modified model of the Mediterranean diet versus a control diet, concluded that the Mediterranean diet is associated with a marked reduction in cardiovascular mortality and in the incidence of cardiovascular complications among patients who have suffered myocardial infarction. However, prior to publication of the PREDIMED (Prevention with Mediterranean Diet) study [22], no randomized clinical trial had investigated whether the effects of the Mediterranean diet are superior to those of a low-fat diet in relation to the primary prevention of cardiovascular diseases. The PREDIMED study [22] was designed to determine whether the Mediterranean diet supplemented with virgin olive oil or nuts prevents the development of major cardiovascular complications in high vascular risk individuals, compared with a low-fat diet. In its initial phase, this study recorded an average increase of 1.8 points on the 14-point scale assessing adherence to the Mediterranean diet, versus an increase of 0.3 points in the control group [28]. During the first year, this implied a decrease in the prevalence of metabolic syndrome and its components and in the incidence of diabetes mellitus [28,29]. Furthermore, after four years of follow-up, the subjects in the intervention groups (Mediterranean diet) showed a 30% decrease in the incidence of major cardiovascular complications versus the control series [30].

### Evaluation of arterial aging

Aging is associated with increased arterial stiffness, which can be evaluated with different clinical and biological tools. The accepted gold standard for assessing arterial stiffness is the carotid-femoral pulse wave velocity (PWV) [31], which has been correlated to increased morbidity-mortality in both patients with cardiovascular disease and in healthy individuals [32]. Central arterial pressure has also been shown to be more strongly correlated to cardiovascular morbidity and mortality than peripheral blood pressure [33]. The central and peripheral augmentation indexes are markers of arterial stiffness which together with aortic systolic pressure and aortic pulse pressure complement the information afforded by pulse wave velocity. The cardio-ankle vascular index (CAVI) also evaluates arterial stiffness, and is able to estimate the risk of atherosclerosis and vascular age. This parameter moreover is independent of arterial pressure [34,35].

### Information technology as a support for improving health

Considering the trend in recent years in the use of mobile telephones, and the recent introduction of smartphones, it can be estimated that within 10 years, a full 80-90% of the population in developed countries will have one of these devices [36], and probably with many more applications than are available today. Information technology is finding many applications in the field of health and Medicine. Recently, short message service (SMS) communication has demonstrated its usefulness in helping people to stop smoking [37]. Smartphone applications designed to assist healthcare personnel both in training and in decision making processes in daily clinical practice and in emergency care are increasing, and there is already some evidence of their usefulness [38]. Such applications have also been used in patient care for monitoring biological parameters [39], detecting falls in the elderly [40], preventing cognitive impairment [41] and monitoring diabetes [42], as well as in cardiac rehabilitation [43] and also in the promotion of physical activity [44] and the management of [45]. Communication based on the use of mobile phones, and especially smartphones, has a strong potential to transform healthcare and clinical interventions in the community [36]. However, its effectiveness needs to be evaluated in multicenter clinical trials, as recommended by the United States Food and Drug Administration (FDA), in a way similarly to what is done with drug substances. A metaanalysis of the use of information technology in dietetic evaluation has concluded that it could improve dietary assessment in some population groups, though improved validity and reliability is required in evaluating micronutrients [46]. Many applications have been developed in the field of health, and specifically in relation to diet and exercise, destined for use by the general population. Although the acceptance and ease of use of such applications has been evaluated among young adults for the registry of eating habits and physical activity [47], they have not been tested in other population types, and little is known of their effectiveness in relation to health outcomes. Few studies have validated these tools, though some investigations are presently in course [48]. Even less work has been done to explore the impact of such applications in relation to increased physical activity and improved eating habits, and hence in terms of improvement of the health of the people who use them [49].

The present study aims to provide evidence of the effect which the new information and communication technologies, and specifically smartphone applications, could have as tools supporting changes in favor of more healthy lifestyles. An evaluation is also made of the effect of such changes upon arterial aging.

### Objectives

The first objective of this study is to develop and validate a smartphone application supporting standardized counseling to increase physical activity and adapt eating habits to the recommendations of the Mediterranean diet. The second and central objective of the study is to quantify the effect attributable to the developed smartphone application in terms of the modification of habits, adherence to the Mediterranean diet, and increased physical activity. Lastly, an evaluation is made of the effect of the modification of habits upon vascular structure and function, and therefore on arterial aging.

## Methods/Design

### Design and setting

This is a randomized, multicenter, double-blind clinical trial involving two parallel groups, designed to evaluate the possible effects of adding an information and communication tool (intervention) in support of behavioral and educational counseling (control) to promote increased physical activity and adaptation of eating habits to the Mediterranean diet.

The study will include 6 groups of the Research Network on Preventive Activities and Health Promotion (REDIAPP) in Bilbao, Cuenca, Zaragoza, Valladolid, Barcelona and Salamanca (Spain), as a continuation of the EVIDENT project developed in 2010–2012.

### Subjects

#### *Study population*

The study population will be selected from the EVIDENT project [7], comprising 1,553 subjects randomly selected in the primary care setting. The study will exclude patients over 70 years of age (due to difficulties in using information and communication tools among such individuals), those unable to do exercise or follow the Mediterranean diet, as well as those subjects who meet any of the exclusion criteria of the EVIDENT project (known coronary or cerebrovascular atherosclerotic disease; heart failure; moderate or severe chronic obstructive pulmonary disease; musculoskeletal disease that limited walking; advanced respiratory, renal, or hepatic disease; severe mental disease; treated oncological disease diagnosed in the 5 years) [7]. The rest of the subjects will be randomised on a centralized basis from Salamanca using the Epidat 4.0 software package to intervention (IG) and control groups (CG) with a ratio of 1/1. Of the 1553 subjects, it is estimated that 10-15% will be excluded; the resulting sample therefore will comprise 1,350 individuals. We estimate that another 10% will reject participation, i.e., we expect to include at least 1,215 subjects in the final sample.

#### *Sample size*

Estimation of sample size has been made for the main study endpoints. Regarding physical exercise, and assuming α = 0.05 and β = 0.10, with a standard deviation (SD) of 154, we would need 1,110 subjects (555 per group) to detect an increase of 30 counts/minute in IG versus CG. In turn, regarding the Mediterranean diet (MD), and assuming α = 0.05 and β = 0.10, with a standard deviation (SD) of 2, we would need 676 subjects (338 per group) to detect an increase of 0.5 points in the MD questionnaire in IG versus CG. The inclusion of 1,215 subjects is considered sufficient to detect clinically relevant differences in the main study endpoints, assuming the cluster effect of the design.

### Variables and measurement instruments

The general and potentially effect-modifying variables, such as age, gender, occupation, smoking, alcohol consumption, personal history and drug use will be documented.

#### *Mobile phone tool for evaluating healthy lifestyles*

**Figure 1:**
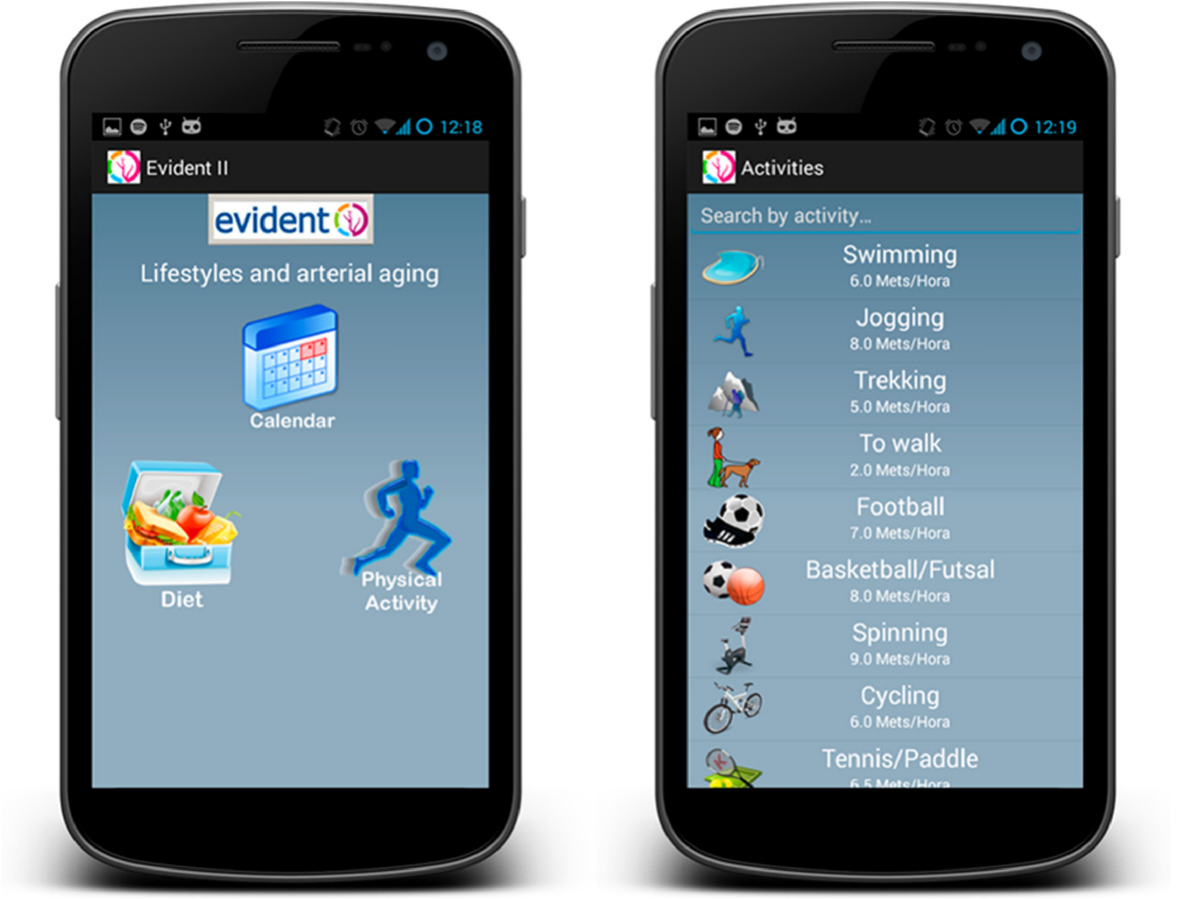
**Smartphone application (APP) EVIDENT II**, **main screen and physical activity.**

**Figure 2:**
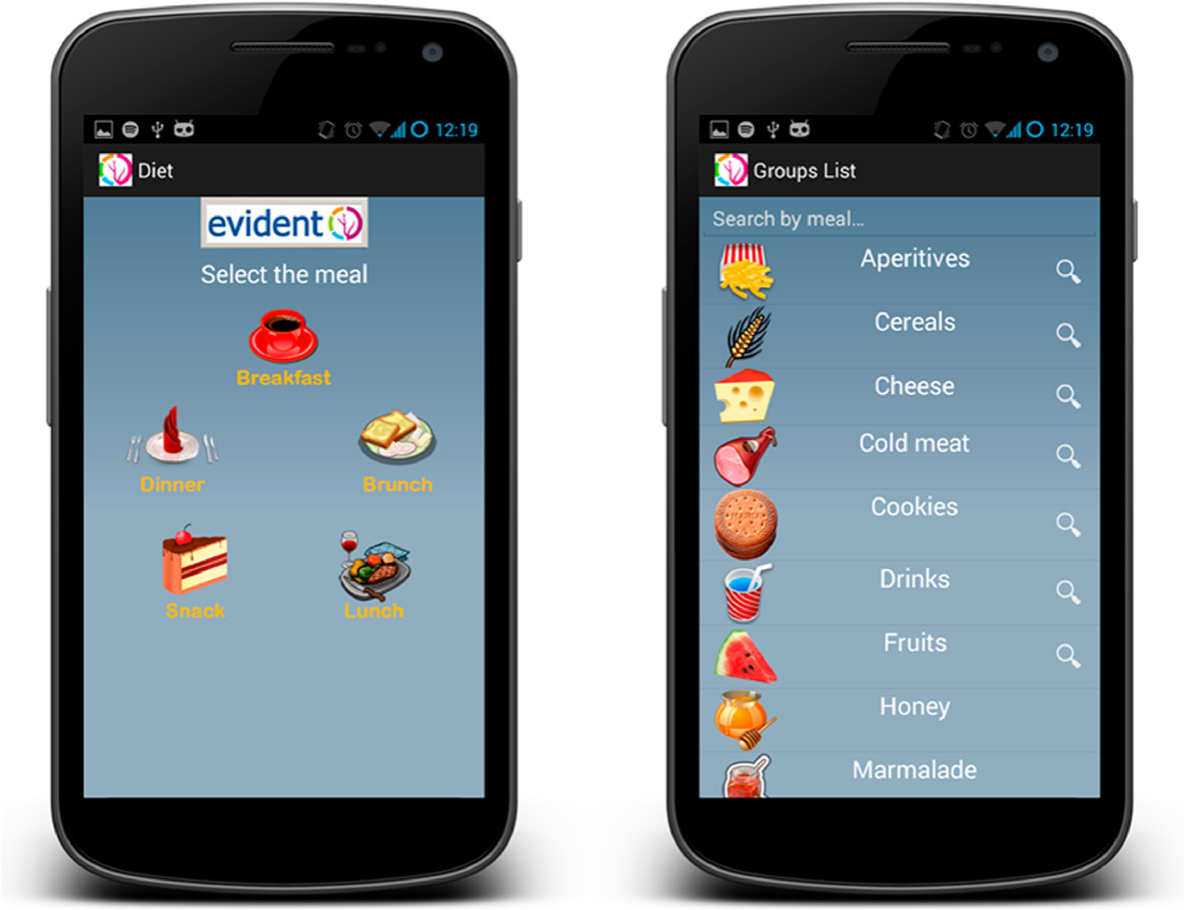
Smartphone application (APP) EVIDENT II, nutrition screens.

The developed tool is the result of an agreement between the company CGB and the GIAPCyL research group of the REDIAPP (RD12/0005/0004), through the *Infosalud Fundation* (Figures 1 and 2). The tool is a smartphone application with a user-friendly environment that is easy for adults to handle. It can be used to quickly evaluate the adaptation of living habits to healthy lifestyle recommendations referred to both eating and physical activity. An evaluation will be made of the quantity and quality of food intake according to standardized references, with the purpose of assessing adaptation of the eating habits of the individual to the Mediterranean diet. Based on adequate proportions of primary food elements, a personalized recommendation will be produced, depending on the entered intake characteristics. The application will generate detailed information on the eating deviations (diet composition and calories), with a view to facilitating changes in habits. Physical activity also will be evaluated, using an accelerometer included in the smartphone application, and which will count the steps taken over a period of 24 hours. The activity reported by the subject during periods in which the device is not used (e.g., swimming and other sports) also will be considered, assessing compliance with the exercise objectives and providing recommendations to increase activity and reach the target of 10,000 steps a day defining an active individual. A balance of activity and diet compliance will be made at the end of the day, and specific recommendations will be produced for the next day/days. The information will be stored in the device and will be downloaded on occasion of the control visits for subsequent analysis.

#### *Physical activity*

Physical activity will be measured by an accelerometer and by the 7-day physical activity recall (PAR). ActiGraph GT3X accelerometers (ActiGraph, Shalimar, FL, USA) will be used to evaluate principal endpoint of psychical activity, which have been previously validated [50-52]. ActiGraph is a monitor that uses a piezoelectric acceleration sensor to filter and convert the signals produced from the sensor in samples collected at a preset frequency in hertz. The samples are summed over a user-specified time sampling interval, called an “epoch”. Output from the ActiGraph is in the form of activity “counts,” where one count is equivalent to 16 milli-g per second, and where g is equal to 9.825 m · s - 2, the acceleration of gravity. Activity “counts” are recorded to the internal memory of accelerometers by converting acceleration units over a given epoch [53]. Subjects will wear the accelerometer fastened with an elastic strap to the right side of the waist for seven consecutive days with habitual physical activity, except for bathing and performing activities in the water. All subjects will be verbally instructed on how to use the accelerometer. The accelerometer will be set to record physical activity data every minute. The MAHUFFE software, available from: http://www.mrc-epid.cam.ac.uk/research/resources/materials-transfer-disclaimer/physical-activity-downloads/ (accessed: 30/12/2013), will be used to analyze the data. Sequences of 10 or more consecutive zero counts will be considered non-wearing time and excluded from the analyses. Inclusion criteria will be a minimum of 4 days of recording, including at least 1 weekend day and at least 600 registered minutes per day. The main outcome variable from the activity monitor will be the average intensity of physical activity (counts/minute), calculated with equal weighting given to each day (regardless of registered time per day). The intensity of physical activity will be determined according to the cut-off points proposed by Freedson [54], sedentary (<100 counts/minute), light (100–1952 counts/minute), moderate (1952–5724 counts/minute) vigorous (>5724 counts/minute) and very vigorous (>9498 counts/minute). Moderate-vigorous activity will be considered as activity accumulated from all bouts lasting at least 1 min.

The 7-day PAR is a general measure of physical activity, which has been recognized as valid and reliable tool in recent years and is widely used in epidemiological, clinical and behavior change studies. It consists of a semi-structured interview (10-15 minutes) in which participants provide an estimate of the number of hours dedicated to physical or occupational activities requiring at least a moderate effort in the past seven days. The categories collected are moderate, vigorous, and very vigorous physical activity. The amount of time dedicated to each activity is multiplied by the mean metabolic equivalents (METs) of each category: light activity 1.5, moderate 4, vigorous 6, and very vigorous 10. The sum of the products of the hours dedicated to each activity and its estimated mean energy expenditure (MET) provides an estimation of the kilocalories per kilogram used per day (kcal*kg-1 * d-1). The dose of physical exercise will be estimated in METs/hour/week and active persons were considered as those doing at least 30 minutes of moderate activity, five days a week, or at least 20 minutes of vigorous activity, 3 days a week or 450 MET · min · wk^-1 ^[12]. Persons not reaching this level of physical activity were considered sedentary.

Questionnaire hours seated (Marshall): Evaluates the hours that the individual is sitting, in their work, in the displacements and at home, during the week and the weekend [55].

#### *Nutrition*

Adherence to the Mediterranean diet, principal endpoint of alimentation, will be measured using the validated 14-point Mediterranean Diet Adherence Screener (MEDAS) [56], developed by the PREDIMED study group. The MEDAS is a valid instrument for rapid estimation of adherence to the Mediterranean diet and may be useful in clinical practice. The 14-item screener includes 12 questions on food consumption frequency and two questions on food intake habits considered characteristic of the Spanish Mediterranean diet. Each question will be scored as 0 or 1. One point will be give for using olive oil as the principal source of fat for cooking, preferring white meat over red meat, or for consuming: 1) 4 or more tablespoons (1 tablespoon = 13.5 g) of olive oil/d; 2) 2 or more servings of vegetables/d; 3) 3 or more pieces of fruit/d; 4) < 1 serving of red meat or sausages/d; 5) < 1 serving of animal fat/d; 6) < 1 cup (1 cup = 100 ml) of sugar-sweetened beverages/d; 7) 7 or more servings of red wine/wk; 8) 3 or more servings of pulses/wk; 9) 3 or more servings of fish/wk; 10) fewer than 2 commercial pastries/wk; 11) 3 or more servings of nuts/wk; or 12) 2 or more servings/wk of a dish with a traditional sauce of tomatoes, garlic and onion. The end score will range from 0 to 14. Adequate adherence to the Mediterranean diet will be assumed when the total score is above or equal to 9 points [56].

The dietary habits of participants and information about dairy products were assessed using a semi-quantitative 137-item food-frequency questionnaire previously validated in Spain [57]. The questionnaire was based on the typical portion sizes that were multiplied by the consumption frequency for each food. This estimated frequency corresponds to the previous year at the time of the interview and it is divided into 9 intake frequencies ranging from never to more than 6 servings/day. This will be used to estimate daily energy intake, essential nutrients, vitamins, fiber, antioxidants and other nutrients.

#### *Laboratory determinations*

Venous blood sampling will be performed between 08:00 and 09:00 hours after the individuals fasted and abstained from smoking and the consumption of alcohol and caffeinated beverages for the previous 12 hours. Fasting plasma glucose, creatinine, uric acid, serum total cholesterol, HDL-cholesterol and triglyceride concentrations will be measured using standard enzymatic automated methods. LDL cholesterol will be estimated by the Friedewald equation when the direct parameter will be not available. Glycated haemoglobin will be measured with an immune-turbidimetric assay. High sensitive C-reactive protein levels and fibrinogen concentrations will be determined by immunoturbidimetric assay. Blood samples will be collected in the respective health centers, and all will be analyzed at the hospital of the city participating in external quality assurance programs of the Spanish Society of Clinical Chemistry and Molecular Pathology.

#### *Anthropometric measurements*

Body weight will be determined on two occasions using a homologated electronic scale (Seca 770; Medical scale and measurement systems, Birmingham, United Kingdom) following due calibration (precision ± 0.1 kg), with the patient wearing light clothing and shoeless. These readings will be rounded to 100 g. Height in turn will be measured with a portable system (Seca 222; Medical scale and measurement systems, Birmingham, United Kingdom), recording the average of two readings, and with the patient shoeless in the standing position. The values will be rounded to the closest centimeter. Body mass index (BMI) will be calculated as weight (kg) divided by height squared (m^2^). A value of > 30 kg/m^2^ will be taken to define obesity. Waist circumference will be measured using a flexible graduated measuring tape with the patient in the standing position without clothing. The upper border of the iliac crests will be located, and the tape will be wrapped around above this point, parallel to the floor, ensuring that it will be adjusted without compressing the skin. Adiposity indices, waist-height and waist-hip, will also be calculated.

#### *Office or clinical blood pressure*

Office blood pressure measurement will involve three measurements of systolic blood pressure (SBP) and diastolic blood pressure (DBP), using the average of the last two, with a validated OMRON model M10-IT sphygmomanometer (Omron Health Care, Kyoto, Japan), by following the recommendations of the European Society of Hypertension [58]. Pulse pressure will be estimated with the mean values of the second and third measurements.

#### *Central blood pressure and radial augmentation index*

Central blood pressure (CBP) and radial augmentation index (rAIx) will be measured with Pulse Wave Application Software (B-pro (A-Pulse)) (Health STATS International) using tonometry to capture the radial pulse and by an equation to estimate central blood pressure (CASP). This device has been validated in asian hypertensives and healthy caucasians [59,60]. rAIx is a measurement taken directly from the late systolic shoulder of the peripheral arterial waveform, and is defined as the ratio of the difference between the 2nd peak and diastolic pressure to the difference between the 1st peak and diastolic pressure [61], it is age-dependent and could be a useful index of vascular aging [62]. rAIx will be calculated as follows: (Second peak systolic blood pressure [SBP2] - diastolic blood pressure [DBP])/(first peak SBP - DBP) × 100 (%) and it will be adjusted for heart rate at 75 bpm and it will be reported as rAIx75. Specific aspects regarding the validity and reliability of the measurement of central blood pressure and augmentation index have been reported elsewhere [60]. In brief, the intra-observer reliability was evaluated in 20 subjects by using the intraclass correlation coefficient (ICC), that showed values of r = 0.971 (95% CI: 0.923 to 0.989) for CASP and 0.952 (95% CI: 0.871 to 0.982) for rAIx. According to the Bland-Altman analysis, the mean difference for intraobserver agreement (95% limits of agreement) were -0.056 (-9.41 to 9.30) for CASP and 2.50 (-14.43 and 19.46) for rAIx. In 104 subjects we examined the agreement of measurements with SphygmoCor and found an ICC for CASP r = 0.972 (95%CI 0.959 to 0.981) and for rAIx r = 0.599 (95% CI: 0.409 to 0.728). In Bland–Altman analysis the mean difference for intraobserver agreement (95% limits of agreement) were 1.47 (0.47 to 2.47) in CASP and 5.85 (1.75 to 9.96) in rAIx.

#### *Assessment of vascular structure by carotid intima media thickness (IMT)*

Carotid ultrasound to assess C-IMT will be performed by two investigators trained for this purpose before starting the study. A Sonosite Micromax ultrasound device paired with a 5–10 MHz multi-frequency high-resolution linear transducer with Sonocal software will be used for performing automatic measurements of carotid IMT in order to optimize reproducibility. Measurements will be made of the common carotid after the examination of a 10 mm longitudinal section at a distance of 1 cm from the bifurcation, performing measurements in the proximal and in the distal wall in the lateral, anterior and posterior projections, following an axis perpendicular to the artery to discriminate two lines, one for the intima-blood interface and the other for the media-adventitious interface. A total of 6 measurements will be obtained of the right carotid and other 6 of the left carotid, using average values (average carotid IMT) and maximum values (maximum carotid IMT) automatically calculated by the software [63]. The measurements will be obtained with the subject lying down, with the head extended and slightly turned opposite to the examined carotid artery. The reliability was evaluated before the study began using the intraclass correlation coefficient, which showed values of 0.974 (95% CI: 0.935 to 0.990) for intra-observer agreement on repeated measurements in 20 subjects, and 0.897 (95% CI:0.740 to 0.959) for inter-observer agreement. According to the Bland-Altman analysis, the mean difference for intraobserver agreement (95% limits of agreement) was 0.022 (95% CI: -0.053 to 0.098) and intra-observer agreement was 0.012 (95% CI: -0.034 to 0.059). The average IMT will be considered abnormal if it measured > 0.90 mm, or if there will be atherosclerotic plaques with a diameter of 1.5 mm or a focal increase of 0.5 mm or 50% of the adjacent IMT [64].

#### *Cardio-ankle vascular index and ankle-brachial index*

Cardio-Ankle Vascular Index (CAVI) and Ankle-brachial index (ABI) will be measured using Vasera device VS-1500® (Fukuda Denshi). For the study, the lowest ankle-brachial index obtained will be considered. The pulse wave velocity (PWV) will be calculated, as well as Cardio-Ankle Vascular Index (CAVI), which gives a more accurate calculation of the atherosclerosis degree. CAVI integrates cardiovascular elasticity derived from the aorta to the ankle pulse velocity through an oscillometric method and it is used as a good measure of vascular stiffness and it doesn’t depend on blood pressure [35]. CAVI values will be automatically calculated by substituting the stiffness parameter β in the following equation to detect the vascular elasticity and the cardio-ankle PWV: Stiffness parameter β = 2ρ x 1/(Ps –Pd) x ln (Ps/Pd) x PWV^2^, where ρ is the blood density, Ps and Pd are SBP and DBP in mmHg, and the PWV is measured between the aortic valve and ankle. The average coefficient of the variation of the CAVI is less than 5%, which is small enough for clinical use and confirm that CAVI has favorable reproducibility [34]. Cardio-Ankle Vascular Index and Ankle-brachial index will be measured at rest.

#### *Analysis of motivation to change*

The model of Prochaska and Diclemente will be used [65]. The motivation stage will be classified on occasion of each interview, based on the following criteria: a) Pre-contemplation: The subjects are unaware that certain behaviors place their health at risk or that they have a health problem; alternatively, they are aware of the existence of a health problem but are reluctant to accept changes in behavior. b) Contemplation: The subjects are aware that certain behaviors place their health at risk or that they have a health problem, and agree to introduce changes within 6 months. c) Determination: The subjects seriously intend to change their behaviour in the near future (within 30 days). d) Action: The subjects are actively working upon changes in behavior that affect health or the identified health problem. e) Maintenance: The subjects routinely adopt the acquired behaviors. Maintenance is considered to have been reached when the new behavior persists for over 6 months. f) Relapse: The subjects start the cycle over again, i.e., they no longer adhere to the desired behavior.

#### *Adherence to the tool*

Adherence will be assessed by means of the Morisky-Green test [66], and by assessing the number and days of recordings in the device.

### Intervention

#### *Common to both groups*

##### 

**Counseling on physical activity** Both groups (control and intervention) will receive counseling on physical activity with a view to favoring compliance with the current recommendations on physical activity in the general population. The intervention has demonstrated its effectiveness in the PEPAF study [14] and in the *Prescribe Vida Saludable* (PSV) program (Osakidetza). Counseling will consist of an individual visit lasting 15 minutes in which an explanation will be given of the health benefits of physical activity, with the recommendation to perform at least 30 minutes of moderate activity 5 days a week, or 20 minutes of vigorous activity three days a week. Counseling will be standardized in both groups, and the subjects will receive an informational brochure (Additional file 1) on the session. The first part (5 min.) will address the recommendations on physical exercise in relation to cardiovascular health. The second part (8 min.) will develop knowledge of the intensity of some specific physical activities such as walking, riding a bicycle, or other activities. The last part (2 min.) will be dedicated to answering questions and doubts. Specific advice will be given to reduce the number of hours spent sitting.

##### 

**Nutritional counseling** Both groups (control and intervention) will receive nutrition counseling aimed at favoring adherence to the MD. This intervention has been shown to be effective in the PREDIMED study [22]. Counseling will consist of an individual visit lasting 15 minutes in which the concepts of the MD will be explained, with insistence upon the importance of complying with each of the recommended points. Counseling will be standardized in both groups, and the subjects will receive an informational brochure (Additional file 1) on the session. The first part (3 min.) will develop the concept of the MD. The second part of the session (10 min.) will focus on each particular recommendation, with brief and clear messages. The last part of the interview (2 min.) will be dedicated to answering questions and doubts.

#### *Specific of the intervention group*

The subjects in the IG will receive a smartphone for three months, corresponding to the intervention period. A first visit lasting 15 minutes will be used to provide training in the use of the device, which should be employed daily for the full three-month period of the intervention. The investigator will instruct the participants on the use of the tool that evaluates food intake, on how to enter the information and receive the recommendations, and on how to use of the accelerometer and read the generated information – with the recommendation to reach a total of 10,000 daily steps. The subjects will be required to daily enter food intake (breakfast, lunch, afternoon snack, and dinner), selecting the dishes and foods from the application menu. Regular physical activity will be recorded with the accelerometer of the device, together with due registry of those activities performed without the smartphone (swimming, football, etc.). Lastly, the final daily summary will be reviewed, with a balance of food intake and physical activity, and the device will offer a recommended plan for the next days, with a view to improving eating habits and increasing physical activity. A new visit will be held one week after supplying the device, in order to confirm that it is being used correctly, and to clarify any possible doubts. The smartphone will be returned after three months, coinciding with the common review visit.

#### *Blinding strategy*

The investigator performing randomization and intervention in the IG will be different from the investigator conducting evaluation and the rest of interventions, blinding will be maintained during the study, and the investigator performing data analysis also will be blinded. Due to the nature of the study, the subjects cannot be blinded to the intervention.

### Project schedule

The project is planned as three yearly periods with subsequent follow-up to evaluate the long-term effects of the intervention. In a previous phase conducted in 2013, the software for the smartphones was developed by the company CGB, and a prior pilot evaluation was carried out in Salamanca among users of both sexes with different ages and cultural levels, to confirm the practical feasibility of the tool and introduce any necessary changes. In the present project, pilot evaluation will be made in 2014–2015, along with recruitment of all the subjects and conduction of the first visits, while in 2015–2016 we will complete the follow-up visits (12 months) – with conclusion of the operative phase of the project on 31 December 2016.

**Figure 3:**
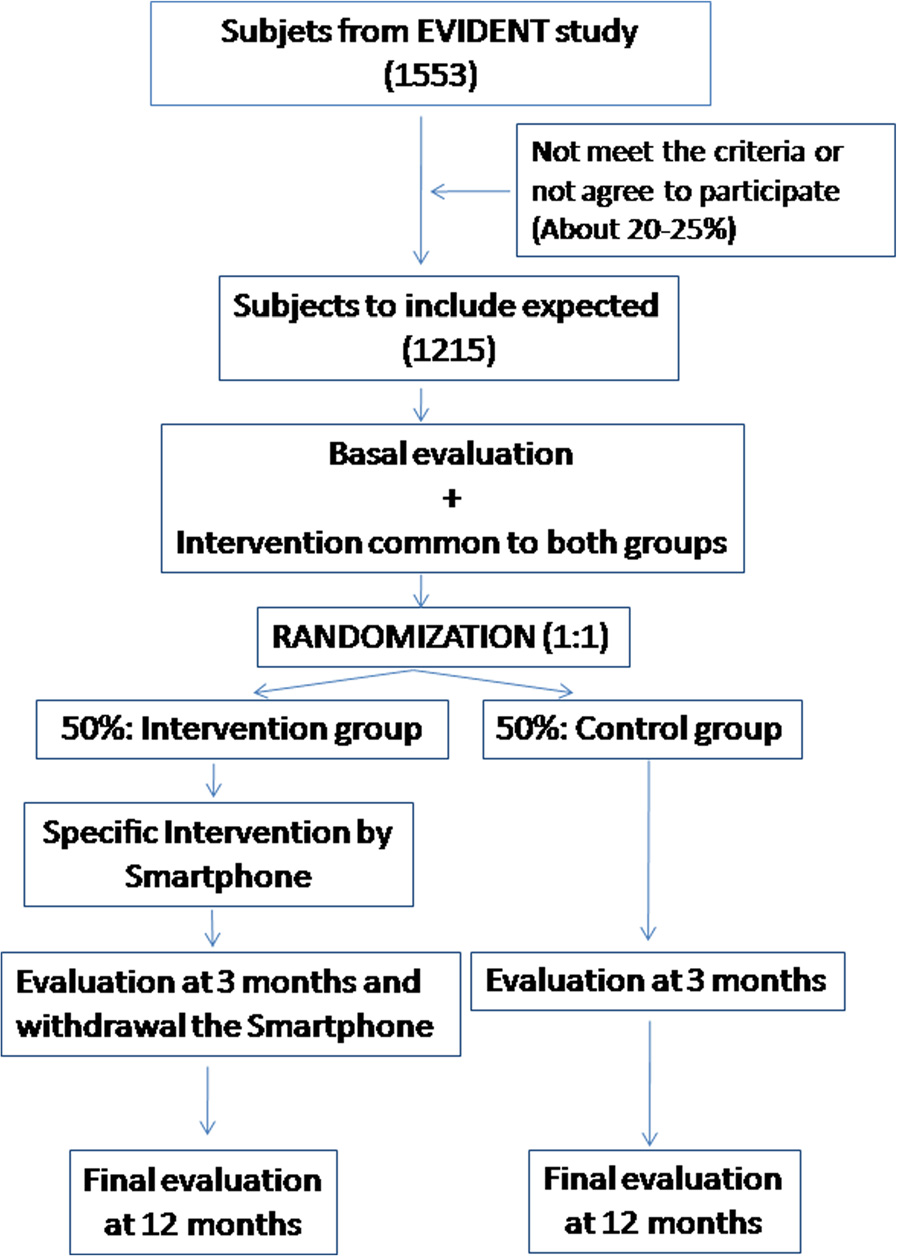
Flow - chart of EVIDENT II clinical trial.

The flow chart (Figure 3) shows the phases of the clinical trial.

### Statistical analysis

The results will be expressed as the mean ± standard deviation for quantitative variables or using the frequency distribution for qualitative variables. Analysis of the results will be made on an intent-to-treat (ITT) basis. Statistical normality will be tested using the Kolmogorov-Smirnov test. Use will be made of the chi-squared test to analyze the association between independent qualitative variables, along with the McNemar test for paired samples. The Student t-test will be used for the comparison of means between two groups, and the paired t-test will be applied to assess changes within one same group. Alternatively, the corresponding nonparametric tests will be used, as required. The relationship between quantitative variables will be analyzed using Pearson’s correlation coefficient or Spearman coefficient in the case of asymmetrically distributed variables. Multivariate linear regression analysis and logistic regression analysis will be used to analyze the variables determining the changes in physical activity and eating habits, and in the arterial stiffness parameters (PWV, AIx and CAVI). In order to analyze the effect of the intervention, comparison will be made of the changes observed in the control group (CG) versus the intervention group (IG), with estimation of the Cohen d statistic, adjusting for the variables that may influence the results. Logistic regression will analyze the odds ratio (OR) for achieving the objectives of diet and exercise compliance. A multilevel analysis will be performed to determine the effect of the different recruitment centers. Gender will be contemplated in the analysis to assess differences between males and females in terms of adherence to the smartphone application, and in the middle- and long-term results. Likewise, an analysis will be made according to the degree of motivation at baseline assessment and adherence to use of the smartphone. The effect of the intervention could be modified by age, gender, cultural and socioeconomic level, body mass index (BMI) and certain disease conditions, as well as by the baseline lifestyles, which will be controlled in the analysis. The contrasting of hypotheses establishes α = 0.05. The IBM-SPSS version 20.0 statistical package will be used throughout.

### Quality control

In order to ensure data quality, the nursing professionals in charge of data collection will receive specific training. Regular external monitoring will then be performed in the six health centers to verify adequate application of methods, both in performing the different examinations and collecting the information.

### Methodological limitations

The study follows all the recommendations of the CONSORT, though in view of the nature of the intervention, the participating subjects will not be blinded to the intervention. Since we are dealing with lifestyle modifications, the analysis of the main results referred to the Mediterranean diet is based on self-declared data, even though use will be made of validated tools and the general food questionnaire, which may serve as quality control. In any case, objective data will be available in the case of physical exercise (accelerometer). Difficulties in using the application may increase the number of dropouts in the intervention group.

### Ethical and legal issues

The study has been approved by the clinical research ethics committee (CEIC) of the healthcare area of Salamanca (“CEIC of Area de Salud de Salamanca”, 21 June 2013), as coordinating center, and by the ethics committees of the collaborating centers (“CEIC of Aragón (CEICA), CEIC of IDIAP Jordi Gol, CEIC of Euskadi (CEIC-E), CEIC of the Area integrada de salud de Talavera de la Reina and CEIC of the Área de Salud de Valladolid Oeste”). Subjects will be required to sign the informed consent prior to inclusion in the study, in accordance with the Declaration of Helsinki [67]. Subjects will be informed of the objectives of the project and of the risks and benefits of the examinations made. None of the examinations pose life-threatening risks for the type of subjects to be included in the study. The study includes the obtainment of biological samples; the study subjects therefore will be informed in detail. The confidentiality of the recruited subjects will be ensured at all times in accordance with the provisions of current legislation on personal data protection (15/1999 of 13 December, LOPD), and the conditions contemplated by Act 14/2007 on biomedical research.

## Discussion

The exponential development of smartphone applications in the field of healthcare has not been accompanied by sufficient scientific evidence of the usefulness of such applications in improving the health of the population. As has already been called upon by different organisms, and more specifically by the FDA, it seems necessary to conduct experimental studies similar to those used in pharmacological treatments, in order to obtain sufficient scientific evidence of the possible usefulness of these tools.

The EVIDENT I study obtained evidence of the association between healthy lifestyles - particularly physical activity and diet – and vascular structure and function. Specifically, less physical activity or prolonged sedentarism was associated to greater aging of the vascular system [10,68].

The EVIDENT II study developed a smartphone application that has already been tested in a prior pilot phase designed to assess its reliability in the recording and analysis of diet and physical activity, as well as in relation to the counseling needed to improve these parameters and come closer to the international recommendations referred to the compliance with the Mediterranean diet and physical activity equivalent to at least 10,000 steps a day.

With this project we hope to demonstrate that regular use of this application for at least three months is able to modify certain habits, resulting in eating habits more in line with the Mediterranean diet, which has already demonstrated its benefits in terms of cardiovascular morbidity-mortality [30]. Likewise, we hope to increase the physical activity of the participants and to raise the proportion of individuals considered to be active, as this should imply multiple improvements in health – as has already been demonstrated in different studies [1-3].

Although over the short term it will be difficult to demonstrate the health benefits of the changes in diet and physical activity, over the middle term we hope to demonstrate that the use of this tool can not only improve living habits but also afford benefits in terms of vascular function - improving the parameters that assess arterial stiffness, such as AIx, CAVI and PWV.

Therefore, the results of this study could lead to a strategy based on the use of new technologies to delay arterial aging, combining increased physical activity and eating habits adapted to the Mediterranean diet. This strategy based on the incorporation of new technologies that are widely disseminated among the population, would result in healthy lifestyles and could constitute a powerful tool against vascular aging.

## Abbreviations

ABI: Ankle-brachial index; Aix: Augmentation index; BMI: Body mass index; CAVI: Cardio-ankle vascular index; CIT: Communication and information technology; DBP: Diastolic blood pressure; PEPAF: Experimental program for Physical Activity Promotion; ESH: European Society of Hypertension; IMT: Intima-media thickness; EVIDENT: Lifestyles and vascular aging; MET: Metabolic equivalent; PREDIMED: Prevention by Mediterranean diet; PWV: Pulse wave velocity; PAR: Physical activity recall; REDIAPP: Research Network on Preventive Activities and Health Promotion; SBP: Systolic blood pressure.

## Competing interests

The authors declare that they have no competing interests.

## Authors’ contributions

Conception of the idea for the study: LG-O and JIR-R. Development of the protocol, organization and funding: LG-O, JIR-R, MÁG-M, JAER-S, JAM-F, DP-A, CM-C, AG-A, NGonzález-Viejo, EI-SN and YS. Writing of the manuscript: JIR-R, LG-O and MAG-M. All the authors have read the draft critically, to make contributions, and have approved the final text.

## Pre-publication history

The pre-publication history for this paper can be accessed here:

http://www.biomedcentral.com/1471-2458/14/254/prepub

## Supplementary Material

Additional file 1 Informational brochure to support the common intervention. Click here for file

## References

[B1] Varo Cenarruzabeitia JJ Martinez Hernandez JA Martinez-Gonzalez MA Benefits of physical activity and harms of inactivity Med Clin (Barc) 2003 121 17 665 672 10.1016/S0025-7753(03)74054-8 14642230

[B2] Myers J Prakash M Froelicher V Do D Partington S Atwood JE Exercise capacity and mortality among men referred for exercise testing N Engl J Med 2002 346 11 793 801 10.1056/NEJMoa011858 11893790

[B3] Whelton SP Chin A Xin X He J Effect of aerobic exercise on blood pressure: a meta-analysis of randomized, controlled trials Ann Intern Med 2002 136 7 493 503 10.7326/0003-4819-136-7-200204020-00006 11926784

[B4] Vaitkevicius PV Fleg JL Engel JH O'Connor FC Wright JG Lakatta LE Yin FC Lakatta EG Effects of age and aerobic capacity on arterial stiffness in healthy adults Circulation 1993 88 4 Pt 1 1456 1462 8403292 10.1161/01.cir.88.4.1456

[B5] Tanaka H Dinenno FA Monahan KD Clevenger CM DeSouza CA Seals DR Aging, habitual exercise, and dynamic arterial compliance Circulation 2000 102 11 1270 1275 10.1161/01.CIR.102.11.1270 10982542

[B6] Lakka TA Laukkanen JA Rauramaa R Salonen R Lakka HM Kaplan GA Salonen JT Cardiorespiratory fitness and the progression of carotid atherosclerosis in middle-aged men Ann Intern Med 2001 134 1 12 20 10.7326/0003-4819-134-1-200101020-00008 11187415

[B7] Garcia-Ortiz L Recio-Rodriguez JI Martin-Cantera C Cabrejas-Sanchez A Gomez-Arranz A Gonzalez-Viejo N Iturregui-San Nicolas E Patino-Alonso MC Gomez-Marcos MA Physical exercise, fitness and dietary pattern and their relationship with circadian blood pressure pattern, augmentation index and endothelial dysfunction biological markers: EVIDENT study protocol BMC Public Health 2010 10 233 10.1186/1471-2458-10-233 20459634 PMC2881095

[B8] Garcia-Ortiz L Recio-Rodriguez JI Schmidt-Trucksass A Puigdomenech-Puig E Martinez-Vizcaino V Fernandez-Alonso C Rubio-Galan J Agudo-Conde C Patino-Alonso MC Rodriguez-Sanchez E Gomez-Marcos MA Relationship between objectively measured physical activity and cardiovascular aging in the general population - The EVIDENT trial Atherosclerosis 2014 233 2 434 440 10.1016/j.atherosclerosis.2014.01.021 24530775

[B9] Patel AV Bernstein L Deka A Feigelson HS Campbell PT Gapstur SM Colditz GA Thun MJ Leisure time spent sitting in relation to total mortality in a prospective cohort of US adults Am J Epidemiol 2010 172 4 419 429 10.1093/aje/kwq155 20650954 PMC3590043

[B10] Recio-Rodriguez JI Gomez-Marcos MA Patino-Alonso MC Romaguera-Bosch M Grandes G Menendez-Suarez M Lema-Bartolome J Gonzalez-Viejo N Agudo-Conde C Garcia-Ortiz L Association of television viewing time with central hemodynamic parameters and the radial augmentation index in adults Am J Hypertens 2013 26 4 488 494 10.1093/ajh/hps071 23467204

[B11] Gomez-Marcos MA Recio-Rodriguez JI Patino-Alonso MC Martinez-Vizcaino V Martin-Borras C de-la-Cal-Dela-Fuente A Sauras-Llera I Sanchez-Perez A Agudo-Conde C Garcia-Ortiz L Relationship between physical activity and plasma fibrinogen concentrations in adults without chronic diseases PLoS ONE 2014 9 2 e87954 10.1371/journal.pone.0087954 24498413 PMC3912191

[B12] Haskell WL Lee IM Pate RR Powell KE Blair SN Franklin BA Macera CA Heath GW Thompson PD Bauman A Physical activity and public health: updated recommendation for adults from the American College of Sports Medicine and the American Heart Association Med Sci Sports Exerc 2007 39 8 1423 1434 10.1249/mss.0b013e3180616b27 17762377

[B13] Hillsdon M Foster C Thorogood M Interventions for promoting physical activity Cochrane Database Syst Rev 2005 1 CD003180 15674903 10.1002/14651858.CD003180.pub2 PMC4164373

[B14] Grandes G Sanchez A Sanchez-Pinilla RO Torcal J Montoya I Lizarraga K Serra J Effectiveness of physical activity advice and prescription by physicians in routine primary care: a cluster randomized trial Arch Intern Med 2009 169 7 694 701 10.1001/archinternmed.2009.23 19364999

[B15] Baker PRA Francis DP Soares J Weightman AL Foster C Community wide interventions for increasing physical activity Cochrane Database Syst Rev 2011 4 CD008366 21491409 10.1002/14651858.CD008366.pub2

[B16] Orrow G Kinmonth AL Sanderson S Sutton S Effectiveness of physical activity promotion based in primary care: systematic review and meta-analysis of randomised controlled trials BMJ 2012 344 e1389 10.1136/bmj.e1389 22451477 PMC3312793

[B17] Hobbs N Godfrey A Lara J Errington L Meyer TD Rochester L White M Mathers JC Sniehotta FF Are behavioral interventions effective in increasing physical activity at 12 to 36 months in adults aged 55 to 70 years? A systematic review and meta-analysis BMC Med 2013 11 1 75 10.1186/1741-7015-11-75 23506544 PMC3681560

[B18] Gilson ND Puig-Ribera A McKenna J Brown WJ Burton NW Cooke CB Do walking strategies to increase physical activity reduce reported sitting in workplaces: a randomized control trial Int J Behav Nutr Phys Act 2009 6 43 10.1186/1479-5868-6-43 19619295 PMC2717045

[B19] De Greef KP Deforche BI Ruige JB Bouckaert JJ Tudor-Locke CE Kaufman JM De Bourdeaudhuij IM The effects of a pedometer-based behavioral modification program with telephone support on physical activity and sedentary behavior in type 2 diabetes patients Patient Educ Couns 2011 84 2 275 279 10.1016/j.pec.2010.07.010 20732776

[B20] Martinez-Gonzalez MA Sanchez-Villegas A The emerging role of Mediterranean diets in cardiovascular epidemiology: monounsaturated fats, olive oil, red wine or the whole pattern? Eur J Epidemiol 2004 19 1 9 13 15012018 10.1023/b:ejep.0000013351.60227.7b

[B21] Fuentes F Lopez-Miranda J Perez-Martinez P Jimenez Y Marin C Gomez P Fernandez JM Caballero J Delgado-Lista J Perez-Jimenez F Chronic effects of a high-fat diet enriched with virgin olive oil and a low-fat diet enriched with alpha-linolenic acid on postprandial endothelial function in healthy men Br J Nutr 2008 100 1 159 165 18275619 10.1017/S0007114508888708

[B22] Estruch R Martinez-Gonzalez MA Corella D Salas-Salvado J Ruiz-Gutierrez V Covas MI Fiol M Gomez-Gracia E Lopez-Sabater MC Vinyoles E Aros F Conde M Lahoz C Lapetra J Saez G Ros E Effects of a Mediterranean-style diet on cardiovascular risk factors: a randomized trial Ann Intern Med 2006 145 1 1 11 10.7326/0003-4819-145-1-200607040-00004 16818923

[B23] Murie-Fernandez M Irimia P Toledo E Martinez-Vila E Buil-Cosiales P Serrano-Martinez M Ruiz-Gutierrez V Ros E Estruch R Martinez-Gonzalez MA Carotid intima-media thickness changes with Mediterranean diet: a randomized trial (PREDIMED-Navarra) Atherosclerosis 2011 219 1 158 162 10.1016/j.atherosclerosis.2011.06.050 21802081

[B24] Trichopoulou A Kouris-Blazos A Wahlqvist ML Gnardellis C Lagiou P Polychronopoulos E Vassilakou T Lipworth L Trichopoulos D Diet and overall survival in elderly people BMJ 1995 311 7018 1457 1460 10.1136/bmj.311.7018.1457 8520331 PMC2543726

[B25] Martinez-Gonzalez MA Fernandez-Jarne E Serrano-Martinez M Marti A Martinez JA Martin-Moreno JM Mediterranean diet and reduction in the risk of a first acute myocardial infarction: an operational healthy dietary score Eur J Nutr 2002 41 4 153 160 10.1007/s00394-002-0370-6 12242583

[B26] Patino-Alonso MC Recio-Rodriguez JI Magdalena Belio JF Colominas-Garrido R Lema-Bartolome J Gomez Arranz A Agudo-Conde C Gomez-Marcos MA Garcia-Ortiz L Factors Associated with Adherence to the Mediterranean Diet in the Adult Population J Acad Nutr Diet 2013 doi:10.1016/j.jand.2013.07.038

[B27] de Lorgeril M Salen P Martin JL Monjaud I Delaye J Mamelle N Mediterranean diet, traditional risk factors, and the rate of cardiovascular complications after myocardial infarction: final report of the Lyon Diet Heart Study Circulation 1999 99 6 779 785 10.1161/01.CIR.99.6.779 9989963

[B28] Salas-Salvado J Fernandez-Ballart J Ros E Martinez-Gonzalez MA Fito M Estruch R Corella D Fiol M Gomez-Gracia E Aros F Flores G Lapetra J Lamuela-Raventos R Ruiz-Gutierrez V Bullo M Basora J Covas MI Effect of a Mediterranean diet supplemented with nuts on metabolic syndrome status: one-year results of the PREDIMED randomized trial Arch Intern Med 2008 168 22 2449 2458 10.1001/archinte.168.22.2449 19064829

[B29] Salas-Salvado J Bullo M Babio N Martinez-Gonzalez MA Ibarrola-Jurado N Basora J Estruch R Covas MI Corella D Aros F Ruiz-Gutierrez V Ros E Reduction in the incidence of type 2 diabetes with the Mediterranean diet: results of the PREDIMED-Reus nutrition intervention randomized trial Diabetes Care 2011 34 1 14 19 10.2337/dc10-1288 20929998 PMC3005482

[B30] Estruch R Ros E Salas-Salvado J Covas MI Pharm D Corella D Aros F Gomez-Gracia E Ruiz-Gutierrez V Fiol M Lapetra J Lamuela-Raventos RM Serra-Majem L Pinto X Basora J Munoz MA Sorli JV Martinez JA Martinez-Gonzalez MA Primary Prevention of Cardiovascular Disease with a Mediterranean Diet N Engl J Med 2013 369 7 676 7 23432189 10.1056/NEJMoa1200303

[B31] Laurent S Cockcroft J Van Bortel L Boutouyrie P Giannattasio C Hayoz D Pannier B Vlachopoulos C Wilkinson I Struijker-Boudier H Expert consensus document on arterial stiffness: methodological issues and clinical applications Eur Heart J 2006 27 21 2588 2605 10.1093/eurheartj/ehl254 17000623

[B32] Mattace-Raso FU van der Cammen TJ Hofman A van Popele NM Bos ML Schalekamp MA Asmar R Reneman RS Hoeks AP Breteler MM Witteman JC Arterial stiffness and risk of coronary heart disease and stroke: the Rotterdam Study Circulation 2006 113 5 657 663 10.1161/CIRCULATIONAHA.105.555235 16461838

[B33] Roman MJ Devereux RB Kizer JR Lee ET Galloway JM Ali T Umans JG Howard BV Central pressure more strongly relates to vascular disease and outcome than does brachial pressure: the Strong Heart Study Hypertension 2007 50 1 197 203 10.1161/HYPERTENSIONAHA.107.089078 17485598

[B34] Shirai K Utino J Otsuka K Takata M A novel blood pressure-independent arterial wall stiffness parameter; cardio-ankle vascular index (CAVI) J Atheroscler Thromb 2006 13 2 101 107 10.5551/jat.13.101 16733298

[B35] Shirai K Hiruta N Song M Kurosu T Suzuki J Tomaru T Miyashita Y Saiki A Takahashi M Suzuki K Takata M Cardio-ankle vascular index (CAVI) as a novel indicator of arterial stiffness: theory, evidence and perspectives J Atheroscler Thromb 2011 18 11 924 938 10.5551/jat.7716 21628839

[B36] Boulos MN Wheeler S Tavares C Jones R How smartphones are changing the face of mobile and participatory healthcare: an overview, with example from eCAALYX Biomed Eng Online 2011 10 24 10.1186/1475-925X-10-24 21466669 PMC3080339

[B37] Free C Knight R Robertson S Whittaker R Edwards P Zhou W Rodgers A Cairns J Kenward MG Roberts I Smoking cessation support delivered via mobile phone text messaging (txt2stop): a single-blind, randomised trial Lancet 2011 378 9785 49 55 10.1016/S0140-6736(11)60701-0 21722952 PMC3143315

[B38] Low D Clark N Soar J Padkin A Stoneham A Perkins GD Nolan J A randomised control trial to determine if use of the iResus(c) application on a smart phone improves the performance of an advanced life support provider in a simulated medical emergency Anaesthesia 2011 66 4 255 262 10.1111/j.1365-2044.2011.06649.x 21401537

[B39] Neubert S Arndt D Thurow K Stoll R Mobile real-time data acquisition system for application in preventive medicine Telemed J E Health 2010 16 4 504 509 10.1089/tmj.2009.0123 20420541

[B40] Yamada M Aoyama T Okamoto K Nagai K Tanaka B Takemura T Using a Smartphone while walking: a measure of dual-tasking ability as a falls risk assessment tool Age Ageing 2011 40 4 516 519 10.1093/ageing/afr039 21593058

[B41] Dufau S Dunabeitia JA Moret-Tatay C McGonigal A Peeters D Alario FX Balota DA Brysbaert M Carreiras M Ferrand L Ktori M Perea M Rastle K Sasburg O Yap MJ Ziegler JC Grainger J Smart phone, smart science: how the use of smartphones can revolutionize research in cognitive science PLoS One 2011 6 9 e24974 10.1371/journal.pone.0024974 21980370 PMC3182196

[B42] Ciemins E Coon P Sorli C An analysis of data management tools for diabetes self-management: can smart phone technology keep up? J Diabetes Sci Technol 2010 4 4 958 960 10.1177/193229681000400427 20663462 PMC2909530

[B43] Worringham C Rojek A Stewart I Development and feasibility of a smartphone, ECG and GPS based system for remotely monitoring exercise in cardiac rehabilitation PLoS One 2011 6 2 e14669 10.1371/journal.pone.0014669 21347403 PMC3036581

[B44] Rabin C Bock B Desired features of smartphone applications promoting physical activity Telemed J E Health 2011 17 10 801 803 10.1089/tmj.2011.0055 22010977

[B45] Gan KO Allman-Farinelli M A scientific audit of smartphone applications for the management of obesity Aust N Z J Public Health 2011 35 3 293 294 10.1111/j.1753-6405.2011.00707.x 21627732

[B46] Ngo J Engelen A Molag M Roesle J Garcia-Segovia P Serra-Majem L A review of the use of information and communication technologies for dietary assessment Br J Nutr 2009 101 Suppl 2 S102 112 19594959 10.1017/S0007114509990638

[B47] Wohlers EM Sirard JR Barden CM Moon JK Smart phones are useful for food intake and physical activity surveys Conf Proc IEEE Eng Med Biol Soc 2009 2009 5183 5186 19964382 10.1109/IEMBS.2009.5333721

[B48] Fukuoka Y Komatsu J Suarez L Vittinghoff E Haskell W Noorishad T Pham K The mPED randomized controlled clinical trial: applying mobile persuasive technologies to increase physical activity in sedentary women protocol BMC Public Health 2011 11 933 10.1186/1471-2458-11-933 22168267 PMC3295748

[B49] Free C Phillips G Felix L Galli L Patel V Edwards P The effectiveness of M-health technologies for improving health and health services: a systematic review protocol BMC Res Notes 2010 3 250 10.1186/1756-0500-3-250 20925916 PMC2976743

[B50] Melanson EL Jr Freedson PS Validity of the Computer Science and Applications, Inc. (CSA) activity monitor Med Sci Sports Exerc 1995 27 6 934 940 7658958

[B51] Matthews CE Keadle SK Sampson J Lyden K Bowles HR Moore SC Libertine A Freedson PS Fowke JH Validation of a Previous-Day Recall Measure of Active and Sedentary Behaviors Med Sci Sports Exerc 2013 45 8 1629 1638 10.1249/MSS.0b013e3182897690 23863547 PMC3717193

[B52] Plasqui G Westerterp KR Physical activity assessment with accelerometers: an evaluation against doubly labeled water Obesity (Silver Spring) 2007 15 10 2371 2379 10.1038/oby.2007.281 17925461

[B53] Chen KY Bassett DR Jr The technology of accelerometry-based activity monitors: current and future Med Sci Sports Exerc 2005 37 11 Suppl S490 500 16294112 10.1249/01.mss.0000185571.49104.82

[B54] Freedson PS Melanson E Sirard J Calibration of the Computer Science and Applications, Inc. accelerometer Med Sci Sports Exerc 1998 30 5 777 781 10.1097/00005768-199805000-00021 9588623

[B55] Marshall AL Miller YD Burton NW Brown WJ Measuring total and domain-specific sitting: a study of reliability and validity Med Sci Sports Exerc 2010 42 6 1094 1102 19997030 10.1249/MSS.0b013e3181c5ec18

[B56] Schroder H Fito M Estruch R Martinez-Gonzalez MA Corella D Salas-Salvado J Lamuela-Raventos R Ros E Salaverria I Fiol M Lapetra J Vinyoles E Gomez-Gracia E Lahoz C Serra-Majem L Pinto X Ruiz-Gutierrez V Covas MI A short screener is valid for assessing Mediterranean diet adherence among older Spanish men and women J Nutr 2011 141 6 1140 1145 10.3945/jn.110.135566 21508208

[B57] Fernandez-Ballart JD Pinol JL Zazpe I Corella D Carrasco P Toledo E Perez-Bauer M Martinez-Gonzalez MA Salas-Salvado J Martin-Moreno JM Relative validity of a semi-quantitative food-frequency questionnaire in an elderly Mediterranean population of Spain Br J Nutr 2010 103 12 1808 1816 10.1017/S0007114509993837 20102675

[B58] O'Brien E Asmar R Beilin L Imai Y Mancia G Mengden T Myers M Padfield P Palatini P Parati G Pickering T Redon J Staessen J Stergiou G Verdecchia P Practice guidelines of the European Society of Hypertension for clinic, ambulatory and self blood pressure measurement J Hypertens 2005 23 4 697 701 10.1097/01.hjh.0000163132.84890.c4 15775768

[B59] Williams B Lacy PS Yan P Hwee CN Liang C Ting CM Development and validation of a novel method to derive central aortic systolic pressure from the radial pressure waveform using an N-point moving average method J Am Coll Cardiol 2011 57 8 951 961 10.1016/j.jacc.2010.09.054 21329842

[B60] Garcia-Ortiz L Recio-Rodriguez JI Canales-Reina JJ Cabrejas-Sanchez A Gomez-Arranz A Magdalena-Belio JF Guenaga-Saenz N Agudo-Conde C Gomez-Marcos MA Comparison of two measuring instruments, B-pro and SphygmoCor system as reference, to evaluate central systolic blood pressure and radial augmentation index Hypertens Res 2012 35 6 617 623 10.1038/hr.2012.3 22297480

[B61] Munir S Guilcher A Kamalesh T Clapp B Redwood S Marber M Chowienczyk P Peripheral augmentation index defines the relationship between central and peripheral pulse pressure Hypertension 2008 51 1 112 118 10.1161/HYPERTENSIONAHA.107.096016 17998476

[B62] Kohara K Tabara Y Oshiumi A Miyawaki Y Kobayashi T Miki T Radial augmentation index: a useful and easily obtainable parameter for vascular aging Am J Hypertens 2005 18 1 Pt 2 11S 14S 15683726 10.1016/j.amjhyper.2004.10.010

[B63] Gomez-Marcos MA Recio-Rodriguez JI Patino-Alonso MC Agudo-Conde C Gomez-Sanchez L Gomez-Sanchez M Rodriguez-Sanchez E Garcia-Ortiz L Protocol for measuring carotid intima-media thickness that best correlates with cardiovascular risk and target organ damage Am J Hypertens 2012 25 9 955 961 10.1038/ajh.2012.72 22717546

[B64] Mancia G Fagard R Narkiewicz K Redon J Zanchetti A Bohm M Christiaens T Cifkova R De Backer G Dominiczak A Galderisi M Grobbee DE Jaarsma T Kirchhof P Kjeldsen SE Laurent S Manolis AJ Nilsson PM Ruilope LM Schmieder RE Sirnes PA Sleight P Viigimaa M Waeber B Zannad F 2013 ESH/ESC Guidelines for the management of arterial hypertension: the Task Force for the management of arterial hypertension of the European Society of Hypertension (ESH) and of the European Society of Cardiology (ESC) J Hypertens 2013 31 7 1281 1357 10.1097/01.hjh.0000431740.32696.cc 23817082

[B65] Tuah NA Amiel C Qureshi S Car J Kaur B Majeed A Transtheoretical model for dietary and physical exercise modification in weight loss management for overweight and obese adults Cochrane Database Syst Rev 2011 10 CD008066 10.1002/14651858.CD008066.pub2 21975777

[B66] Morisky DE Green LW Levine DM Concurrent and predictive validity of a self-reported measure of medication adherence Med Care 1986 24 1 67 74 10.1097/00005650-198601000-00007 3945130

[B67] WMA World Medical Association Declaration of Helsinki: ethical principles for medical research involving human subjects JAMA 2013 310 20 2191 2194 24141714 10.1001/jama.2013.281053

[B68] Garcia-Ortiz L Recio-Rodriguez JI Puig-Ribera A Lema-Bartolome J Ibanez-Jalon E Gonzalez-Viejo N Guenaga-Saenz N Agudo-Conde C Patino-Alonso MC Gomez-Marcos MA Blood Pressure Circadian Pattern and Physical Exercise Assessment by Accelerometer and 7-Day Physical Activity Recall Scale Am J Hypertens 2013 doi: 10.1093/ajh/hpt159

