# Supplementary material for: Effectiveness of a smartphone application for improving healthy lifestyles, a randomized clinical trial (EVIDENT II): study protocol
Source: BMC Public Health. 2014 Mar 15;14:254. doi: 10.1186/1471-2458-14-254 (PMC4003852; doi:10.1186/1471-2458-14-254)

Colaboran:

redIAPP
Red de Investigación en Actividades Preventivas y Promoción de la Salud

IDIAP Jordi Gol

La Alamedilla
Centro de salud
Unidad de Investigación

IBSAL
Instituto de Investigación Biomédica de Salamanca

infoSalud

CGB

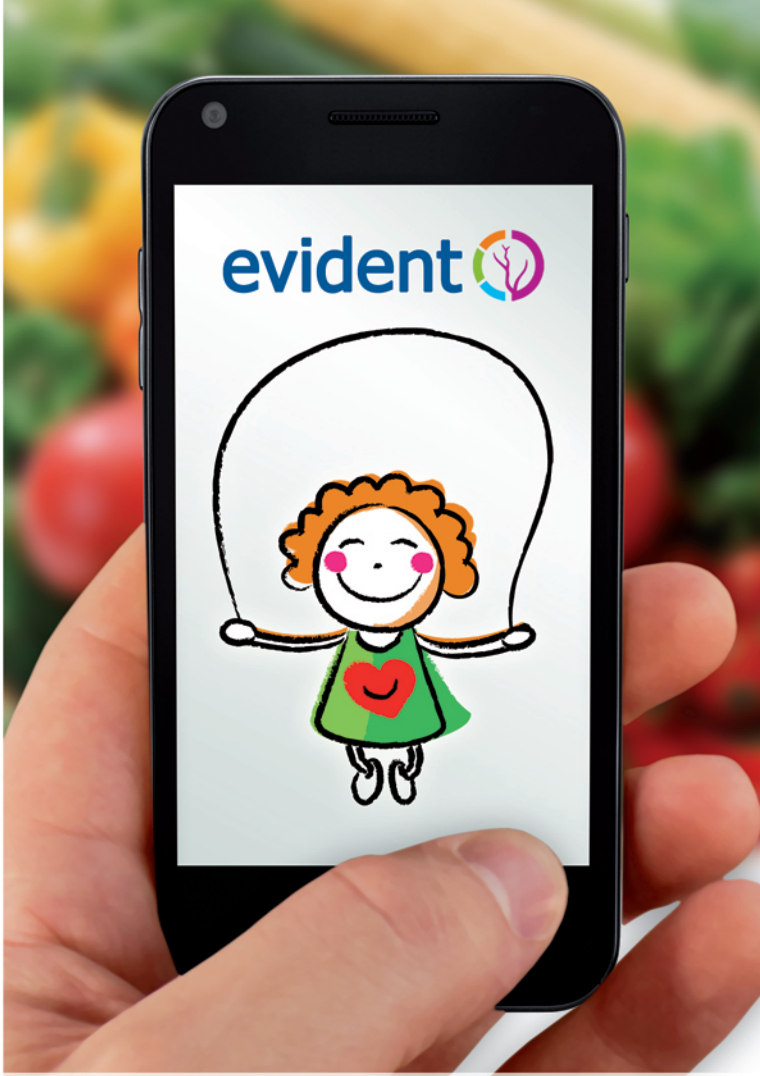

EFECTIVIDAD DE UNA APLICACIÓN PARA SMARTPHONE EN LA MEJORA DE ESTILOS DE VIDA SALUDABLES

ENSAYO CLÍNICO ALEATORIO (EVIDENT II)

Financia: Instituto de Salud Carlos III
y Gerencia Regional de Salud de Castilla y León

Es bien sabido que en los países ribereños del mar Mediterráneo ha habido tradicionalmente una baja frecuencia de enfermedades del corazón y de muertes por esta causa, junto con una larga esperanza de vida. Se cree que esto es debido, entre otros factores, a los hábitos alimentarios tradicionales de las gentes que viven en la zona del Mediterráneo.

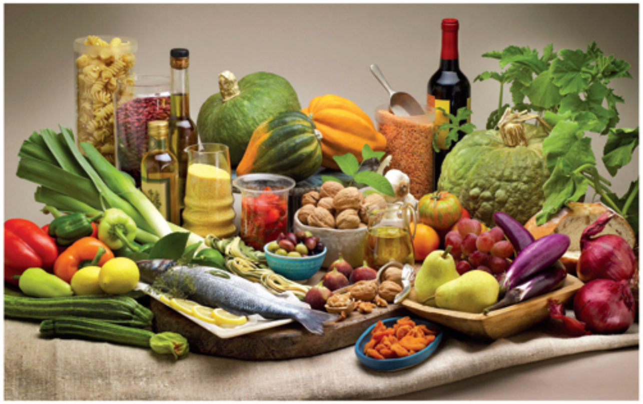

La dieta mediterránea se caracteriza por tener como base de la alimentación el aceite de oliva y los cereales (trigo), aunque a nivel nutricional destacan más alimentos: fruta fresca, verduras, hortalizas, frutos secos, legumbres, una moderada cantidad de vino tinto y una alimentación en la que predomine el pescado sobre la carne.

RECOMENDACIONES PARA SEGUIR UNA DIETA MEDITERRÁNEA

- Utilice aceite de oliva para cocinar y aliñar los alimentos
- Consuma 2 o más raciones al día de verduras y hortalizas (1 como mínimo cruda en ensalada)
- Tome 3 o más piezas de fruta al día (incluyendo zumos naturales)
- Consuma legumbres al menos 3 veces por semana
- Consuma pescados o mariscos al menos 3 veces a la semana (1 como mínimo de pescado azul)
- Tome frutos secos al menos 3 veces por semana
- Consuma con mayor frecuencia carnes blancas (aves sin piel o conejo) que carnes rojas, embutidos u otras carnes procesadas.
- Aderece los platos de pasta, arroz, y verduras con sofritos de tomate, ajo, cebolla o puerro al menos 2 veces a la semana.
- Limite a menos de una vez a la semana el consumo de los siguientes alimentos y bebidas:
  - Nata, mantequilla o margarina.
  - Bebidas azucaradas (refrescos)
  - Repostería o bollería industrial, patatas de bolsa y alimentos o platos precocinados.

Los beneficios de la actividad física, tanto a nivel biológico, como psicológicos, van desde una mejor calidad de vida, a la reducción de los riesgos asociados a la hipertensión, obesidad y diabetes, o a la prevención y control de enfermedades cardiovasculares, osteoporosis, ansiedad, depresión y algunos cánceres.

El tiempo necesario depende de la intensidad del esfuerzo

	Ejercicio Moderado (30 - 60 minutos)	Ejercicio Vigoroso (20 - 30 minutos)
Actividad	Caminar a paso rápido<br>Natación<br>Bicileta<br>Bailar	Footing<br>Aerobic<br>Fútbol, Baloncesto,<br>Tenis ...
Frecuencia	5 a 7 veces por semana	3 o más veces por semana

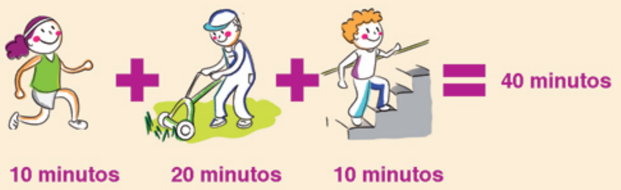

Supplement: Additional file 1 — Informational brochure to support the common intervention. [file 1471-2458-14-254-S1.pdf]
